# Validity of Estimation of Pelvic Floor Muscle Activity from Transperineal Ultrasound Imaging in Men

**DOI:** 10.1371/journal.pone.0144342

**Published:** 2015-12-07

**Authors:** Ryan E. Stafford, Geoff Coughlin, Nicholas J Lutton, Paul W. Hodges

**Affiliations:** 1 The University of Queensland, Centre for Clinical Research Excellence in Spinal Pain, Injury and Health, School of Health and Rehabilitation Sciences, Brisbane, Australia; 2 Department of Urology, Royal Brisbane and Women’s Hospital, Brisbane, Australia; 3 Department of Colorectal Surgery, Princess Alexandra Hospital, Brisbane, Australia; Northwestern University Feinberg School of Medicine, UNITED STATES

## Abstract

**Purpose:**

To investigate the relationship between displacement of pelvic floor landmarks observed with transperineal ultrasound imaging and electromyography of the muscles hypothesised to cause the displacements.

**Materials and Methods:**

Three healthy men participated in this study, which included ultrasound imaging of the mid-urethra, urethra-vesical junction, ano-rectal junction and bulb of the penis. Fine-wire electromyography electrodes were inserted into the puborectalis and bulbocavernosus muscles and a transurethral catheter electrode recorded striated urethral sphincter electromyography. A nasogastric sensor recorded intra-abdominal pressure. Tasks included submaximal and maximal voluntary contractions, and Valsalva. The relationship between each of the parameters measured from ultrasound images and electromyography or intra-abdominal pressure amplitudes was described with nonlinear regression.

**Results:**

Strong, non-linear relationships were calculated for each predicted landmark/muscle pair for submaximal contractions (R^2^–0.87–0.95). The relationships between mid-urethral displacement and striated urethral sphincter electromyography, and bulb of the penis displacement and bulbocavernosus electromyography were strong during maximal contractions (R^2^–0.74–0.88). Increased intra-abdominal pressure prevented shortening of puborectalis, which resulted in weak relationships between electromyography and anorectal and urethravesical junction displacement during all tasks.

**Conclusions:**

Displacement of landmarks in transperineal ultrasound imaging provides meaningful measures of activation of individual pelvic floor muscles in men during voluntary contractions. This method may aid assessment of muscle function or feedback for training.

## Introduction

Ultrasound imaging (US) can visualise movement of pelvic floor structures in men[1–5] and women[6–9] during voluntary contractions and other tasks (e.g. coughing [10]) to investigate pelvic floor muscle activation. This non-invasive measure has great potential to provide a method for widespread clinical and experimental evaluation of pelvic floor muscle control in a range of conditions of continence that affect up to 40% of men at some stage in their life [11](e.g. post-prostatectomy incontinence). This technique relies on a predictable relationship between movements measured with US imaging and muscle activation. There are two issues. First, multiple muscles move pelvic structures. Although a relationship between specific muscles and movements has been inferred from anatomy[4, 5, 10], this requires clarification. Second, the relationship between muscle activation and movement is complex (e.g. muscle activation occurs during lengthening and shortening) and it is necessary to determine whether and under what circumstances movement of anatomical landmarks reflects activation. Previous work highlights that the relationship between ultrasound measures and EMG differs between muscles and ultrasound measures cannot be used to infer activity in all situations [12]. Resolution of the issues related to use of ultrasound imaging to interpret muscle activation from pelvic floor muscles in men requires simultaneous recording of US and electromyography (EMG) of each pelvic floor muscle.

Observation of movement of pelvic structures (e.g. urethrovesical junction[3] and mid-urethra[4, 5]) with transabdominal or transperineal US has been used to infer activation of either specific muscles of the pelvic floor or the whole muscle group in general. The transperineal approach enables visualisation of movement of multiple pelvic structures simultaneously. In men, a method has been described to quantify movement at specific pelvic landmarks that are predicted to relate to specific muscles[5]. Based on anatomy, it was proposed that dorsal movement at mid-urethra (MU) is caused by striated urethral sphincter (SUS) activation, ventral/cranial movement of the urethrovesical junction (UVJ) and anorectal junction (ARJ) is explained by puborectalis (PR) activation; and bulbocavernosus (BC) activation compresses the bulb of the penis (BP). The predicted interpretation of specific movements to infer activation of specific muscles requires validation by simultaneous investigation of EMG of each muscle and the motions measured from US images.

The relationship between the amplitude of movement at each landmark and muscle activation also require investigation. US measures of changes in muscle morphology relate in a non-linear manner to changes in EMG during low-level isometric contractions[12–14]. The non-linearity of this relationship is largely explained by the properties of the non-contractile muscle attachments. At low contraction levels, tendon stiffness is low and small increases in muscle activity cause relatively large changes in muscle architecture. As tendon stiffness increases with increasing muscle activity, the potential for change of muscle architecture is reduced. The amplitude and direction of movement of pelvic landmarks will also depend on the balance between amplitude of pelvic floor muscle activation and the intra-abdominal pressure (IAP)[15]. If muscle force exceeds the resistance from IAP, the muscle will shorten and pelvic structures are likely to move as predicted. Yet, if the force of IAP exceeds the muscle force, then the muscle will lengthen (contract eccentrically). Observation of lengthening would be difficult to interpret as it may occur with or without pelvic floor muscle activation. Although some data of this complex relationship are available for motion of the bladder base in women[15], this has not been studied in men and consequently no data are available for MU and BP motion, yet these components may be less affected by IAP as BC is external to the abdominal cavity and the expected motion of MU is dorsal (similar to the direction of motion induced by IAP) rather than cranial (motion opposed by IAP).

Estimation of pelvic floor muscle activity using US imaging represents a potentially valuable non-invasive tool for assessment and training of these muscles in clinical practice and for large scale studies of continence disorders in men. The first aim of this study was to quantify the characteristics of each landmark displacement/pelvic floor muscle EMG activity relationship using a method previously reported for investigations of other axial and limb muscles. The second aim was to investigate whether movement at an individual landmark specifically relates to the EMG activity of the muscle hypothesised to cause the movement. The third aim was to investigate the influence of IAP on the displacement/EMG activity relationship at for each landmark/muscle pair. Based on previous reports of limb and axial skeletal muscles[12] we hypothesized that EMG and landmark displacement would be related in a non-linear manner if shortening was not opposed by elevated IAP.

## Materials and Methods

### Participants

This study was approved by the University of Queensland Medical Research Ethics Committee, approval #2010000545, and conducted in accordance with the principles expressed in the Declaration of Helsinki. All participants provided written consent. Three healthy men (age: 26–44 years) with no history of urological disorders participated. Participants took part in another study[16].

### Measurement

Participants sat reclined on a plinth with knees extended and back rest at approximately 30° from vertical. US was recorded in video format with a transducer (M7C, GE Healthcare, Australia) placed in the mid-sagittal plane on the perineum. A foot switch triggered each US recording and was used to synchronise US with IAP and EMG. A naso-gastric pressure transducer (CTG-2, Gaeltec Ltd, UK) recorded IAP. PR and BC EMG was recorded with intramuscular electrodes fabricated from pairs of fine-wires (Teflon coated, stainless steel wire, diameter– 75μm). With US guidance, an experienced colorectal surgeon inserted the electrodes through the perineum into PR in a cranial direction just left of the anus, and into BC in a ventral direction at the base of the penis. SUS EMG was recorded using a transurethral surface electrode[17] which was self-inserted by the participant after detailed instruction. One participant requested assistance with catheterisation from a supervising urologist. Pelvic floor muscle EMG was amplified 2000x (NL844, Digitimer Ltd, UK), bandpass filtered between 10–2000 Hz (NL125, Digitimer Ltd, UK) and recorded at 10 KHz with a Power1401 analogue to digital converter and Spike2 software (Cambridge Electronic Design, UK).

### Procedure

Three tasks were performed to investigate the relationship between pelvic floor movement, EMG activity and IAP. These were: (i) sub-maximal contraction of the pelvic floor muscles to with an effort of 3 out of 10 estimated using a modified Borg scale in response to the verbal instruction to “shorten the penis” (three repetitions); (ii) maximum voluntary contraction (MVC) of the pelvic floor muscles (three repetitions); and (iii) a Valsalva manoeuvre with effort increasing from rest to maximum over ~5s (two repetitions).

### Data analysis

**Fig 1:**
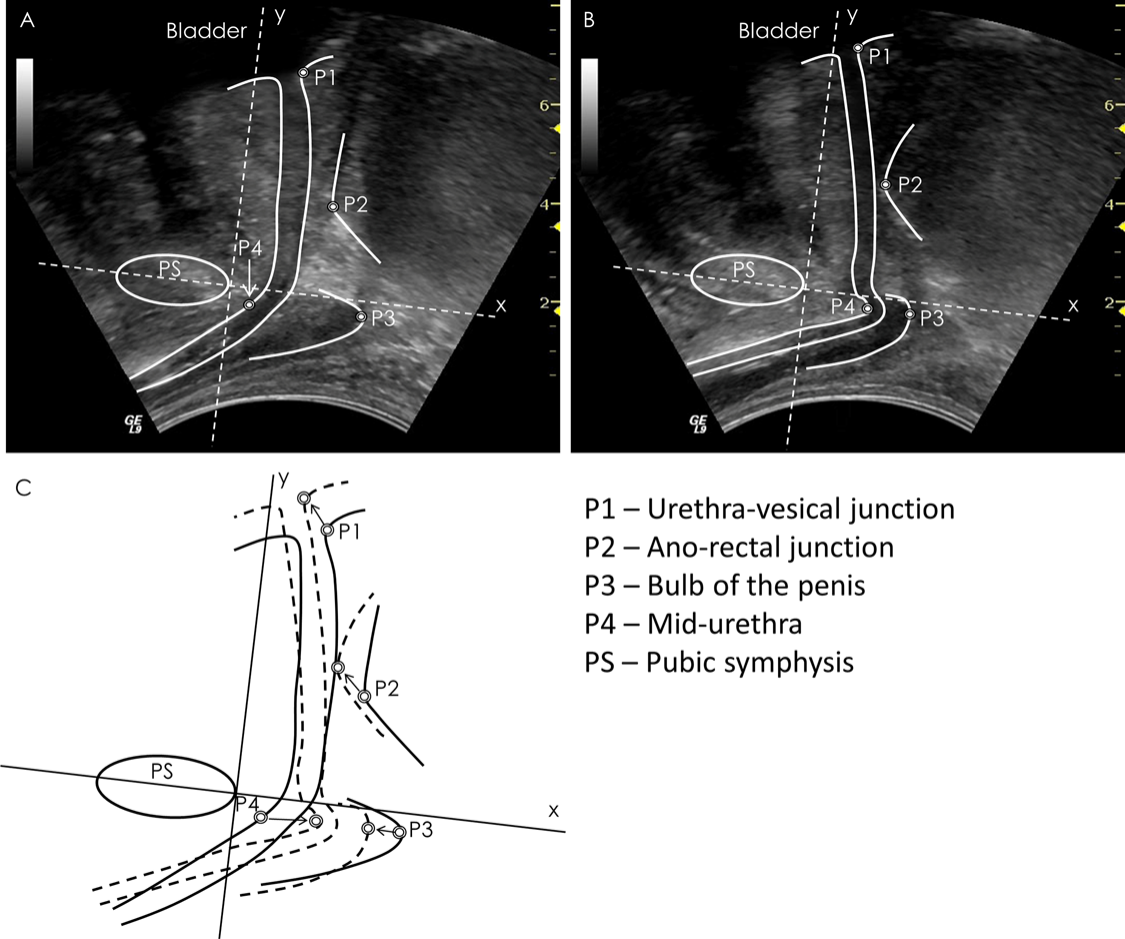
Method for calculation of pelvic floor displacements. Representative transperineal ultrasound images recorded in the (A) relaxed and (B) contracted states with pelvic structure borders and points of interest superimposed on the images. (C) Combined borders from the two images to indicate change in position. The circles indicate the points of interest used for displacement calculations, and the arrows indicate direction of displacement with voluntary contraction.

Displacement of anatomical landmarks was calculated frame-by-frame from the US data from the time of onset of movement to the peak displacement using a method that referenced MU, ARJ, UVJ and BP landmarks to the pubic symphysis (Fig 1) [5]. The root-mean-squared (RMS) amplitudes of SUS, PR and BC EMG and mean IAP amplitude were calculated over 100-ms windows corresponding with the timing of the US frames. EMG and IAP data were normalised to MVC. Because of high variation of US measures during MVC efforts (largely as a consequence of the effect of IAP on landmark motion) displacement data were normalised to the displacement amplitude achieved when EMG was recorded at ~50% MVC for each muscle[12] for comparison between tasks. The displacement amplitude at ~50% MVC was estimated from an exponential curve fitted to the data using the method described below.

### Statistical analysis

The relationships between EMG and displacement data were calculated using nonlinear regression as described previously[12]. The exponential function *D* = *a* × (1 − *e* ^−*EMG* / *b*^) was fitted to the data with the Marquardt-Levenberg algorithm using Matlab software (r2011b, The Mathworks, USA). “*D*” is the displacement, “*a*” is asymptote value for “*D*” (i.e. the value of D if the curve was extended to infinity), and “*b*” determines with curvature of the relationship. The “*a*”, “*b*” and associated R^2^ values were averaged over the repetitions for each condition (Sub-maximal contraction, MVC, Valsalva) and used for comparison.

To address aim one (whether changes in displacement are related to EMG amplitude) the mean “*a*” and “*b*” values were calculated for the group by averaging across participants to understand the representative curvature of each relationship between landmark displacement and activation of the corresponding muscle. To address aim two (whether movement of anatomical landmarks is related to EMG of the muscles predicted to induce the movement), the relationship between displacement and EMG amplitude for each possible landmark/muscle pair was investigated through comparison of Pearson’s R^2^. To address aim three data were considered qualitatively/quantitatively with respect to the effect of IAP on the EMG-displacement relationship.

## Results

**Fig 2:**
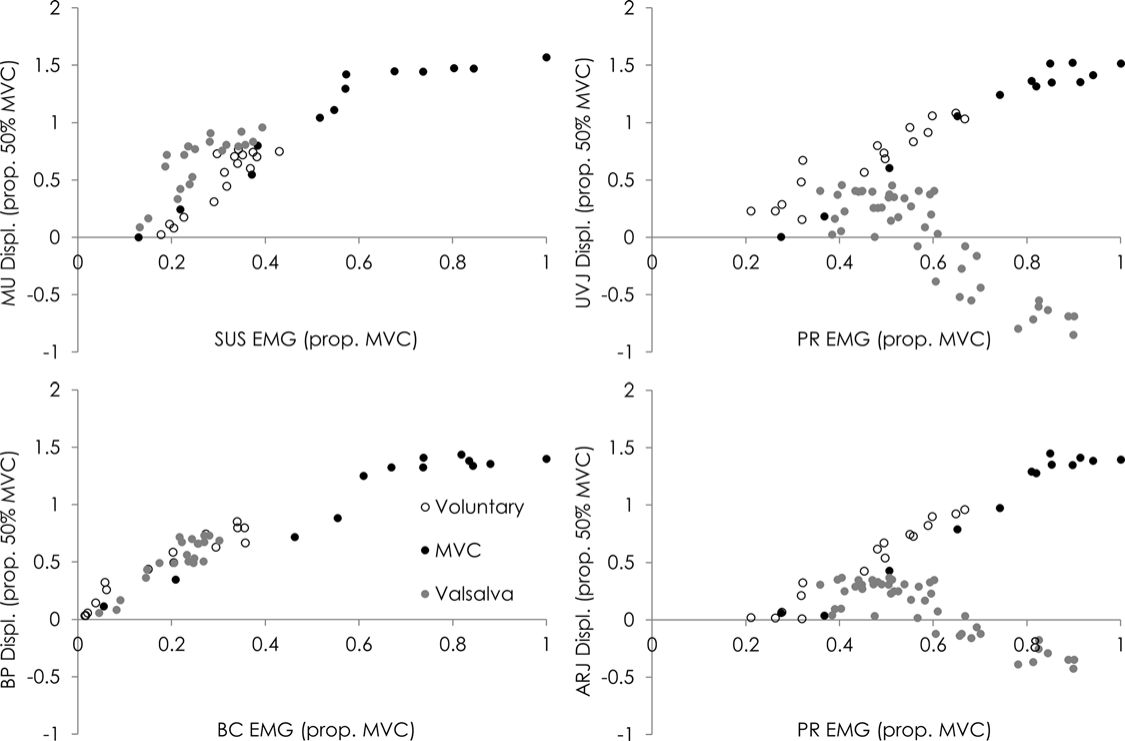
Data of the relationships between landmark displacement and muscle activity for a representative participant for each task; sub-maximal voluntary contraction, maximal voluntary contraction (MVC) and Valsalva. MU–mid-urethra, UVJ–urethra-vesical junction, ARJ–ano-rectal junction, BP–bulb of penis, SUS–striated urethral sphincter, PR–puborectalis, BC–bulbocavernosus.

**Fig 3:**
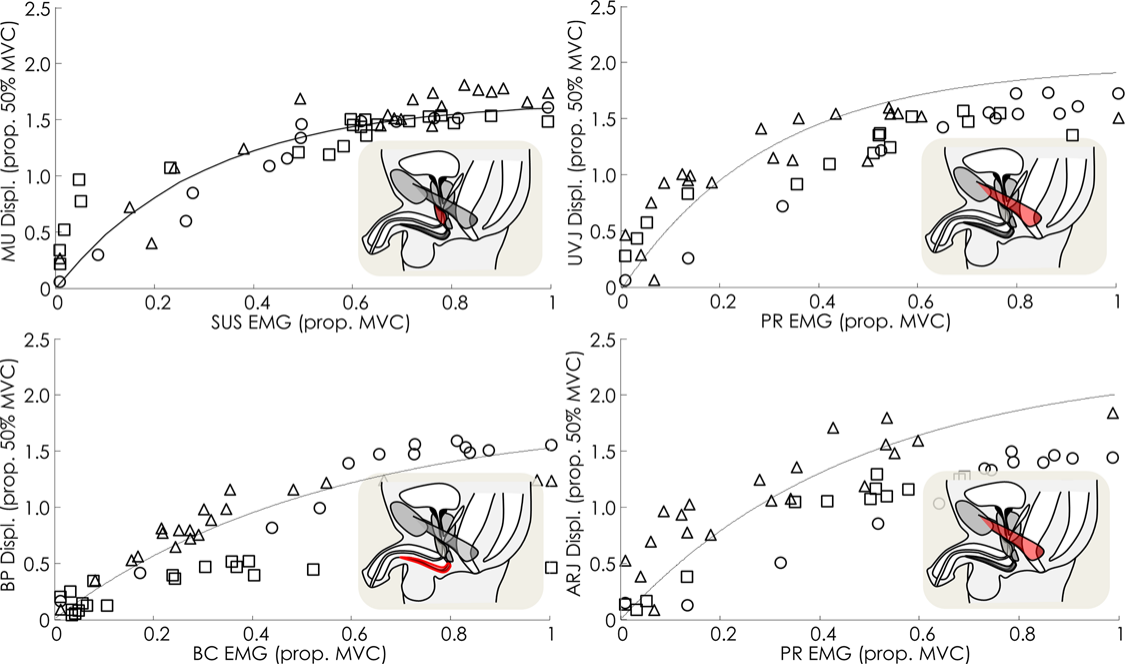
Individual (shapes) and group data (lines) of the relationships between displacement of pelvic floor landmarks observed with transperineal US imaging and activity of the muscles predicted to cause the displacement. Lines are generated using the mean R^2^, asymptote and curvature for the group. MU–mid-urethra, UVJ–urethra-vesical junction, ARJ–ano-rectal junction, BP–bulb of penis, SUS–striated urethral sphincter, PR–puborectalis, BC–bulbocavernosus.

Fig 2 shows the relationship between EMG and displacement measured from ultrasound imaging for a representative participant during each task (shown only for US measures considered to be responsible for each movement, see below). With respect to aim one, as predicted from studies of other muscles the relationship between movement of each landmark and EMG was non-linear. The relationship was characterised by a large initial displacement coupled with a relatively small increase in EMG from rest, followed by a phase of limited displacement despite increasing EMG (Group and individual data for MVC task are shown in Fig 3). Group asymptote (a: value of the ultrasound measure if the curve was extended to infinity), curvature of the relationship (b) and R^2^ values for each landmark/EMG relationship are shown in Table 1. In general, optimal curve-fitting results in an asymptote that lies within a range that is possible according to the measure in question (e.g. for MU this would be in the range of 1.0–5.2 mm).

**Table 1:** Mean asymptote (“*a*”), curvature (“*b*”) and R^2^ values for each comparison of landmark displacement and EMG amplitude for sub-maximal contraction.

	MU			UVJ			ARJ			BP		
	*a*	*b*	R^2^	*a*	*b*	R^2^	*a*	*b*	R^2^	*a*	*b*	R^2^
SUS EMG	**2.4**	**0.2**	**0.88**	3.8	0.3	0.78	425.8	246.3	0.66	103.5	106.5	0.63
PR EMG	296.3	370.7	0.78	**2.0**	**0.3**	**0.89**	**2.3**	**0.5**	**0.88**	1378.7	1100.0	0.81
BC EMG	529.1	271.8	0.73	23.1	10.1	0.82	180.0	112.2	0.78	**1.9**	**0.5**	**0.87**

With respect to aim 2, when participants voluntarily contracted their pelvic floor muscles with a sub-maximal effort (EMG range: [all muscles] 49–69% MVC), each landmark (MU, ARJ, UVJ, BP) moved in the direction that has been predicted from anatomy to reflect muscle shortening (SUS, PR, PR and BC, respectively), and the movement amplitude at each landmark was more strongly correlated with the change in EMG of the muscle predicted to cause the movement (R^2^ range: [all participants and landmarks] 0.87–0.95), than the change in EMG of the other muscles (Table 1). This pattern of greatest correlation coefficient for the relationship between EMG of the muscle predicted to be responsible for each movement than the other muscles was also identified for the maximal pelvic floor muscle contraction (Table 2) and Valsalva (Table 3).

**Table 2:** Mean asymptote (“*a*”), curvature (“*b*”) and R^2^ values for each comparison of landmark displacement and EMG amplitude for maximal voluntary contraction.

	MU			UVJ			ARJ			BP		
	*a*	*b*	R^2^	*a*	*b*	R^2^	*a*	*b*	R^2^	*a*	*b*	R^2^
SUS EMG	**1.6**	**0.3**	**0.88**	808.8	689.3	0.82	690.3	591.0	0.77	223.9	250.3	0.80
PR EMG	124.4	0.5	0.74	**2.3**	**0.3**	**0.83**	**2.3**	**0.7**	**0.86**	498.6	264.2	0.75
BC EMG	1.6	0.7	0.77	1.9	0.7	0.87	2.3	1.1	0.86	**1.6**	**0.7**	**0.83**

**Table 3:** Mean asymptote (“*a*”), curvature (“*b*”) and R^2^ values for each comparison of landmark displacement and EMG amplitude for Valsalva,

	MU			UVJ			ARJ			BP		
	*a*	*b*	R^2^	*a*	*b*	R^2^	*a*	*b*	R^2^	*a*	*b*	R^2^
SUS EMG	**3.7**	**0.1**	**0.83**	-1784.1	1928.1	0.74	-2.0	-0.1	0.76	2.5	0.7	0.76
PR EMG	5.8	0.3	0.72	**-1.3**	**0.1**	**0.70**	**-1.8**	**0.1**	**0.71**	4.3	0.2	0.59
BC EMG	2.1	0.2	0.83	-0.5	1204.8	0.59	1968.9	-853.1	0.63	**1.3**	**0.2**	**0.82**

Bold numbers—pelvic landmark predicted to have the strongest relationship to each muscle’s activity based on anatomy and predicted direction of muscle shortening.

MU–mid-urethra, UVJ–urethra-vesical junction, ARJ–ano-rectal junction, BP–bulb of the penis, SUS–striated urethral sphincter, PR–puborectalis, BC—bulbocavernosus

Data show greater R^2^, plus asymptote and curvature values that are more representative of the values described for other skeletal muscles (range of curvatures for other muscles—0.04–0.28[12]) when the regression line is fitted to the displacement and EMG considered to represent the same muscle (indicated in grey in the Table 1). This provides evidence of specificity of the relationship between muscle activation and movement of the landmark moved by that muscle. In all repetitions of sub-maximal contraction, IAP remained low (range peak amplitude—11–24 cmH_2_O).

**Fig 4:**
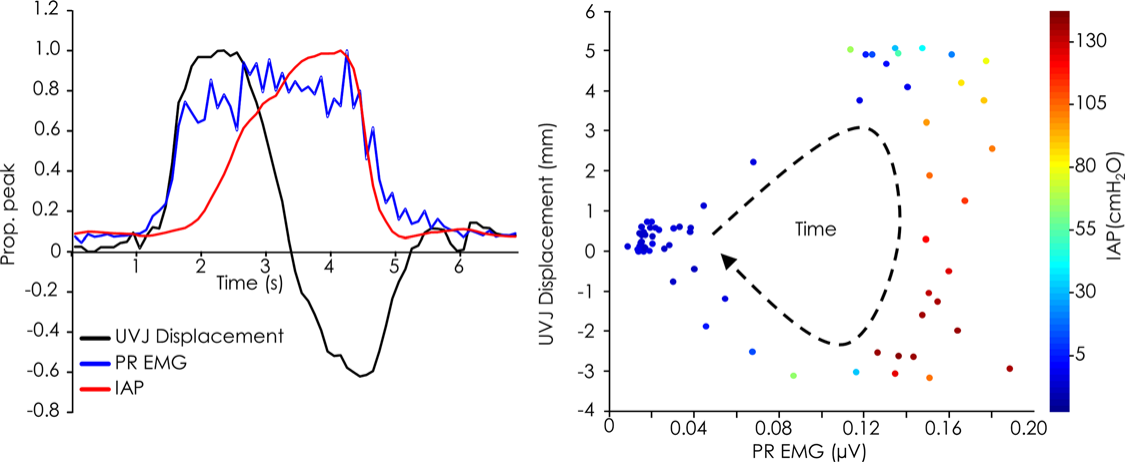
The relationship between displacement at the urethra-vesical junction (UVJ), puborectalis (PR) EMG and intra-abdominal pressure (IAP) for an individual participant during a repetition of the MVC task. Data normalised to peak are shown (left) in addition to the corresponding raw data (right).

During MVC efforts, the non-linear relationships between BP displacement and BC EMG, and between MU displacement and SUS EMG were remained strong. The parameters of the non-linear regression were similar those reported for the sub-maximal voluntary contraction (Table 2). In contrast, relationships between UVJ and ARJ displacement and PR EMG were poor. This was explained by the complex interaction with IAP. The features of this interaction varied between participants and repetitions and data were considered for trials individually. Fig 4 shows an example of the relationship between PR EMG, UVJ displacement and IAP. In this example IAP began to rise early with activation from rest and reached a peak of 131 cmH_2_O. The initial phase involved muscle shortening (ventral/cranial displacement) and a relationship between EMG and displacement that approximated that demonstrated for the sub-maximal contraction task. As IAP increased the UVJ displaced caudally, consistent with lengthening of the muscle as a consequence of the downward force of IAP. This was observed on two occasions; the first and second repetitions of MVC for participant 1 when IAP reached 163 and 131 cmH_2_O, respectively. The asymptote, curvature and R^2^ values from these two MVC efforts could not be calculated. However, when peak IAP was below 75 cmH_2_O (Participant 1, repetition 3; Participants 2&3, all repetitions), the relationship between movement at UVJ and ARJ and PR EMG was strong (R^2^ range: 0.69–0.96) and displayed similar asymptote and curvature values to those described for the sub-maximal contraction (Table 2).

During Valsalva, participants initiated the task slowly from rest over ~5 s and generated peak IAP amplitude in the range of 172–206 cmH_2_O. As with the submaximal and maximal voluntary contractions, MU and BP displaced in a direction indicative of muscle shortening, despite increasing IAP, and was related in a non-linear manner with SUS and BC EMG, respectively (R^2^ range: 0.75–0.90). However, the mean asymptote for MU/SUS EMG was 3.68 and greater than that for MVC (1.62) and sub-maximal contractions (2.39). This would imply exaggeration of dorsal MU displacement by IAP (both IAP and SUS move the MU in this direction) (Fig 2). The parameters of the relationship between BP/BC EMG remained consistent across tasks and which implies independence from effects of IAP. Increased IAP caused movement at UVJ and ARJ in a direction indicative of lengthening despite an increase in PR EMG (Fig 2). As a consequence, the asymptote for the UVJ/PR and ARJ/PR relationships was negative. Although this implies displacement and activation are related during eccentric contraction PR, its interpretation depends on concurrent measurement of IAP and PR activation.

## Discussion

These data provide insight into the interpretation of pelvic floor muscle activity from displacement of anatomical landmarks on transperineal US images. First, displacement of each anatomical pelvic landmark was more strongly related to contraction of the muscles hypothesised to be responsible for the movement than the other muscles (MU vs. SUS muscle; BP vs. BC muscle; UVJ/ARJ vs. PR muscle). Second, movement of the pelvic landmarks was related in a non-linear manner to the amplitude of EMG recorded from the relevant muscle during sub-maximal contractions. The parameters of the non-linear regression lines were more characteristic of values derived for axial/limb muscles when considered for the specific relationships between each landmarks and the corresponding muscle, than the relationship with the other muscles. Third, when IAP increased, activation of PR could not be accurately interpreted from displacement of the UVJ or ARJ because the muscle lengthens despite EMG activity. BP and MU motion retained a strong relationship to EMG despite elevated IAP.

### Relationship between EMG and displacement of pelvic landmarks

Consistent with previous data for other muscles, the relationship between landmark displacement and muscle activity was non-linear. The relationship was characterised by 2 variables, the asymptote and curvature. The asymptote refers to the predicted displacement when the relationship with EMG is extended until infinity, and the curvature of the exponential function influences the predicted amplitude of displacement (until the asymptote is reached). The curvature of the relationships identified in this study (MVC: 0.33–0.67) are greater than values reported for skeletal muscles of the limbs and abdomen (0.04–0.28)[12]. This indicates that from rest to sub-maximal contraction, the amplitude of landmark displacement is less per unit EMG increase than the measures of muscle change used to estimate contraction of other skeletal muscles. There are several possible explanations. The initial phase of shortening of limb/axial muscles is considered to be mediated by extensibility of the tendinous/fascial muscle attachments. A resting muscle, with slackened fascial/teninous attachment[12, 14] can undergo large initial displacement from a relatively small increase in muscle activity (i.e. small curvature value). As the pelvic floor muscles have no/limited tendinous/fascial attachment, this would contribute relatively less to initial displacement for these muscles. Further, tonic activity is often reported for some pelvic floor muscles (e.g. levator ani[18]) further limiting initial length change. A further issue to consider is that whereas the parameters of the regression line were using individual data points averaged across participants for the previous study, here we calculated the regression separately for each participant[12].

### Factors affecting the relationship between muscle activation and displacement of pelvic landmarks

Several physiological mechanisms will influence the relationship between EMG and landmark displacement. These include the dependence of force production on muscle length (length-tension relationship)[19] and the rate of length change (force-velocity relationship)[20]. Further, the amplitude and direction of displacement will depend on forces that oppose muscle shortening. Maximum displacement would be achieved if EMG activity is high and resistance to shortening (e.g. IAP) is low. Muscle activation would not be easily interpreted from US if IAP opposed the muscle shortening; if IAP exceeded muscle activation the muscle would lengthen despite activation (eccentric contraction). Interpretation of activity of pelvic floor muscles from US, using the relationships identified here, is only accurate when muscle force exceeds IAP, and the muscle shortens (concentric contraction). The present data of pelvic floor muscle EMG and landmark displacement show that contractions were associated with either lengthening or shortening of the muscles. Displacement representative of shortening occurred at all landmarks during the sub-maximal tasks, when IAP was low. This enabled accurate interpretation of muscle activity. As IAP increased in some repetitions of maximal pelvic floor muscle contraction and all Valsalva tasks, PR underwent lengthening. Thus, in these tasks it was impossible to interpret PR activation from US. In contrast, SUS and BC shortened despite increased IAP. This is likely explained by inability for IAP to oppose BC, as it is located external to the abdominal cavity, and SUS, as its direction of motion is congruent with the influence of IAP. With reference to the latter, dorsal MU motion related to SUS activity was exaggerated by IAP during Valsalva. This may limit interpretation of SUS from US in specific situations.

### Clinical significance

The findings of this study have potential implications for investigation and management of pelvic floor dysfunction. EMG measures such as those used in this study have limited clinical utility and will not be able to be applied to large populations. Non-invasive methods that are clinically viable are necessary to enable detailed analysis of pelvic floor muscle function in a range of conditions. These data show that transperineal US can be used to interpret activation of individual pelvic floor muscles. Investigations of symptomatic men with US may provide new insight into dysfunction, and provide specific targets for treatment. Conservative treatment of post-prostatectomy incontinence has typically included pelvic floor muscle exercise programmes that encourage activation of the anal sphincter[21–23]. Given the evidence of lack of efficacy of current methods[24], new approaches should be considered. The use of US to as a feedback to aid retraining of activation of pelvic floor muscles relevant to maintenance of urinary continence (e.g. SUS) is a possible solution.

### Methodological considerations

Although a small sample size was used because of the invasive nature of the EMG recording methods, consistent relationships between displacement and EMG for each participant were observed. As it remains unknown how landmark displacement relates to urethral pressure, further work is required to understand the effect of each movement on continence. Although strong relationships were observed between landmark displacement and the muscles predicted to cause the movements, we must consider the potential for muscles not recorded in this study to influence displacement. Evaluation of landmark displacement with electrical stimulation of individual muscles would help confirm the observations of this study.

## Conclusions

Movement of pelvic landmarks observed with transperineal US is non-linearly related to the EMG amplitude of the muscles hypothesised to induce the movement. Transperineal US can be used to interpret the activation of individual male pelvic floor muscles during voluntary contractions. However, this relationship is not ideal for all muscles in all contexts as a result of the impact of elevated IAP on potential motion of some landmarks. This method is likely to be useful for assessment or feedback of muscle activation in men with a range of conditions of continence that are highly prevalent and make a major impact on the quality of life of affected men.

## References

[1] Davis SN, Morin M, Binik YM, Khalife S, Carrier S. Use of pelvic floor ultrasound to assess pelvic floor muscle function in Urological Chronic Pelvic Pain Syndrome in men. J Sex Med. 2011;8:3173–80. 10.1111/j.1743-6109.2011.02452.x 21883952

[2] Geraerts I, Van Poppel H, Devoogdt N, Van Cleynenbreugel B, Joniau S, Van Kampen M. Prospective evaluation of urinary incontinence, voiding symptoms and quality of life after open and robot-assisted radical prostatectomy. BJU Int. 2013;112:936–43. 10.1111/bju.12258 23937206

[3] Nahon I, Waddington G, Adams R, Dorey G. Assessing muscle function of the male pelvic floor using real time ultrasound. Neurourol Urodyn. 2011;30:1329–32. 10.1002/nau.21069 21563212

[4] Stafford RE, Ashton-Miller JA, Constantinou CE, Hodges PW. Novel insight into the dynamics of male pelvic floor contractions through transperineal ultrasound imaging. J Urol. 2012;188:1224–30. 10.1016/j.juro.2012.06.028 22902016 PMC4106154

[5] Stafford RE, Ashton-Miller JA, Constantinou CE, Hodges PW. A New Method to Quantify Male Pelvic Floor Displacement From 2D Transperineal Ultrasound Images. Urology. 2013;81:685–9. 10.1016/j.urology.2012.11.034 23332998 PMC4123322

[6] Dietz HP. Pelvic floor ultrasound: a review. Am J Obstet Gynecol. 2010;202:321–34. 10.1016/j.ajog.2009.08.018 20350640

[7] Dietz HP, Wilson PD, Clarke B. The use of perineal ultrasound to quantify levator activity and teach pelvic floor muscle exercises. Int Urogynecol J Pel. 2001;12:166–9. 10.1007/s001920170059 11451004

[8] Lovegrove Jones RC, Peng Q, Stokes M, Humphrey VF, Payne C, Constantinou CE. Mechanisms of pelvic floor muscle function and the effect on the urethra during a cough. Eur Urol. 2010;57:1101–10. 10.1016/j.eururo.2009.06.011 19560261 PMC2889228

[9] Peng Q, Jones R, Shishido K, Constantinou CE. Ultrasound evaluation of dynamic responses of female pelvic floor muscles. Ultrasound Med Biol. 2007;33:342–52. 17210220 10.1016/j.ultrasmedbio.2006.08.020 PMC1993910

[10] Stafford RE, Mazzone SB, Ashton-Miller JA, Constantinou CE, Hodges PW. Dynamics of male pelvic floor muscle contraction observed with transperineal ultrasound imaging differ between voluntary and evoked coughs. J Appl Physiol. 2014;116:953–60. 10.1152/japplphysiol.01225.2013 24526580 PMC4132566

[11] Sladden MJ, Hughes AM, Hirst GH, Ward JE. A community study of lower urinary tract symptoms in older men in Sydney, Australia. Aust N Z J Surg. 2000;70:322–8. 10830592 10.1046/j.1440-1622.2000.01738.x

[12] Hodges PW, Pengel LH, Herbert RD, Gandevia SC. Measurement of muscle contraction with ultrasound imaging. Muscle Nerve. 2003;27:682–92. 12766979 10.1002/mus.10375

[13] Koppenhaver SL, Hebert JJ, Parent EC, Fritz JM. Rehabilitative ultrasound imaging is a valid measure of trunk muscle size and activation during most isometric sub-maximal contractions: a systematic review. Aust J Physiother. 2009;55:153–69. 19681737 10.1016/s0004-9514(09)70076-5

[14] Herbert RD, Moseley AM, Butler JE, Gandevia SC. Change in length of relaxed muscle fascicles and tendons with knee and ankle movement in humans. J Physiol. 2002;539:637–45. 11882694 10.1113/jphysiol.2001.012756 PMC2290150

[15] Junginger B, Baessler K, Sapsford R, Hodges PW. Effect of abdominal and pelvic floor tasks on muscle activity, abdominal pressure and bladder neck. Int Urogynecol J. 2010;21:69–77. 10.1007/s00192-009-0981-z 19730763

[16] Stafford RE, Ashton-Miller JA, Constantinou C, Coughlin G, Lutton NJ, Hodges PW. Pattern of activation of pelvic floor muscles in men differs with verbal instructions. Neurourol Urodyn. Epub 2015 Mar 1. 10.1002/nau.22745 25727781

[17] Stafford RE, Sapsford R, Ashton-Miller J, Hodges PW. A novel transurethral surface electrode to record male striated urethral sphincter electromyographic activity. J Urol. 2010;183:378–85. 10.1016/j.juro.2009.08.105 19914647

[18] Peschers UM, Vodusek DB, Fanger G, Schaer GN, DeLancey JO, Schuessler B. Pelvic muscle activity in nulliparous volunteers. Neurourol Urodyn. 2001;20:269–75. 11385693 10.1002/nau.1004

[19] Gordon AM, Huxley AF, Julian FJ. The variation in isometric tension with sarcomere length in vertebrate muscle fibres. J Physiol. 1966;184:170–92. 5921536 10.1113/jphysiol.1966.sp007909 PMC1357553

[20] Wilkie DR. The relation between force and velocity in human muscle. J Physiol. 1949;110:249–80. 15406429 10.1113/jphysiol.1949.sp004437 PMC1392741

[21] Dorey G, Glazener C, Buckley B, Cochran C, Moore K. Developing a pelvic floor muscle training regimen for use in a trial intervention. Developing a pelvic floor muscle training regimen for use in a trial intervention. 2009;95:199–209. 10.1016/j.physio.2009.03.003 19635340

[22] Floratos DL, Sonke GS, Rapidou CA, Alivizatos GJ, Deliveliotis C, Constantinides CA, et al Biofeedback vs verbal feedback as learning tools for pelvic muscle exercises in the early management of urinary incontinence after radical prostatectomy. BJU Int. 2002;89:714–9. 11966630 10.1046/j.1464-410x.2002.02721.x

[23] Glazener C, Boachie C, Buckley B, Cochran C, Dorey G, Grant A, et al Urinary incontinence in men after formal one-to-one pelvic-floor muscle training following radical prostatectomy or transurethral resection of the prostate (MAPS): two parallel randomised controlled trials. Lancet. 2011;378:328–37. 10.1016/S0140-6736(11)60751-4 21741700

[24] Campbell SE, Glazener CM, Hunter KF, Cody JD, Moore KN. Conservative management for postprostatectomy urinary incontinence. Cochrane Database Syst Rev. 2012;1:CD001843 10.1002/14651858.CD001843.pub4 22258946

